# Transmission potential of African, Asian and American Zika virus strains by *Aedes aegypti* and *Culex quinquefasciatus* from Guadeloupe (French West Indies)

**DOI:** 10.1080/22221751.2019.1615849

**Published:** 2019-05-20

**Authors:** Lyza Hery, Antoine Boullis, Christelle Delannay, Anubis Vega-Rúa

**Affiliations:** Institute Pasteur of Guadeloupe, Laboratory of Vector Control research, Unit Transmission Reservoir and Pathogens Diversity, Les Abymes, France

**Keywords:** *Aedes aegypti*, *Culex quinquefasciatus*, Zika virus, Guadeloupe, vector competence

## Abstract

Zika virus (ZIKV) is an arbovirus that has dramatically spread in South America and the Caribbean regions since 2015. The majority of vector incrimination studies available for ZIKV showed that *Aedes aegypti* mosquitoes are important vectors for this virus. However, several reports suggest that *Culex quinquefasciatus* mosquitoes may be implicated in ZIKV transmission in certain urban settings. In the present study, we evaluated the vector competence for ZIKV of *Cx. quinquefasciatus* and *Ae. aegypti* mosquitoes from Guadeloupe using African, American and Asian strains. The results demonstrated that *Cx. quinquefasciatus* is refractory to ZIKV infection whatever the strain tested at 7, 14 or 21 days post-infection (dpi), while ZIKV transmission was recorded in *Ae. aegypti* for all the three strains. The African ZIKV strain was better transmitted by *Ae. aegypti* (∼ 50% mean transmission efficiency) and with a shorter incubation period (7 dpi) when compared to the Asian and American strains (<14% transmission efficiency; incubation period of 14–21 dpi). Taken together, these results suggest that only *Ae. aegypti* mosquitoes are involved in urban ZIKV transmission in Guadeloupe and highlight a higher infectiousness of the African ZIKV strain in this mosquito species when compared to the Asian and American ones.

## Introduction

Zika virus (ZIKV) is a single-stranded, positive RNA arbovirus belonging to the *Flaviviridae* family (*Flavivirus* genus) that can cause neurological complications [1]. Phylogenetic studies have identified three major ZIKV lineages: African, Asian and American corresponding with an initial circulation in East Africa and the subsequent spread of the virus in West Africa, Asia and the Americas [2]. ZIKV is mainly transmitted to humans in urban settings by mosquitoes belonging to the *Aedes* genus, such as *Aedes aegypti* and *Aedes albopictus* [3–10]. In Guadeloupe, a French island in the Caribbean with a population of about 400,000 persons, a ZIKV outbreak occurred in 2016 involving 30,500 clinically suspected cases [11]. The mosquito *Ae. aegypti* is suspected to be the sole vector of ZIKV during the outbreak, as *Ae. albopictus* has not been reported yet in the island. Nevertheless, to date there are no reports of field investigations incriminating *Ae. aegypti* from Guadeloupe and the vector competence studies conducted on local *Ae. aegypti* populations have not proven transmission by the species, as ZIKV was not detected in the saliva of the analysed mosquitoes [3,12]. This information is crucial to demonstrate the role of *Ae. aegypti* as ZIKV vector in Guadeloupe, as well as to evaluate the levels of transmission that can be supported by this species that is permanently implanted on the island.

In addition, ZIKV has been detected in *Culicidae* species belonging to other genera than *Aedes* [5,7] and experimental transmission have been proven for some of them [13,14]. Such is the case for *Culex quinquefasciatus*, one of the most abundant mosquitoes in tropical and subtropical urban areas, including Guadeloupe, where the species can share habitats with *Aedes* spp [15,16]. This mosquito is a vector of several pathogens, including flaviviruses genetically related to ZIKV such as West Nile and St. Louis encephalitis viruses [17]. Locally, West Nile virus has been detected in Guadeloupe in horses since 2002 [18]. Even if there is a lack of evidence for natural *Cx. quinquefasciatus* infections associated with titers compatible with ZIKV transmission [7] the results obtained by several vector competence assays conducted on the species continuously nourish the controversy: the majority of studies showed inability for this species to be infected by ZIKV or detail the impermeability of the salivary gland barrier [19,20], while few reports reveal the presence of infectious ZIKV particles in salivary glands or saliva of this mosquito [21,22]. Therefore, the possibility of *Cx. quinquefasciatus* implication in the transmission of ZIKV in Guadeloupe should be evaluated, as the involvement of both *Ae. aegypti* and *Cx. quinquefasciatus* mosquitoes as vectors may affect the vector control strategies to be implemented in the island. In addition to differences in control measures, multiple genera of mosquitoes transmitting ZIKV may also reflect an increased risk for humans.

The objectives of this study were (i) to determine if *Ae. aegypti* and *Cx. quinquefasciatus* from Guadeloupe could have been involved in ZIKV transmission during the past outbreak, and (ii) to evaluate the transmission levels of different ZIKV lineages that can be ensured by local mosquito populations to assess the transmission dynamics of these strains. For these purposes, we performed vector competence assays on field-collected *Ae. aegypti* and *Cx. quinquefasciatus* from Guadeloupe using three ZIKV strains from different lineages (African, Asian and American).

## Material and methods

### Ethics statement

The rabbit blood used for all the experiments was graciously provided by the French Agricultural Research Center for International Development (CIRAD) in Guadeloupe in the frame of a local collaboration. The animals used for blood collection are held in an Animal Experimentation Establishment (EEA) of CIRAD approved under No. C-971-18-02 the 15th March 2017. This EEA (number 69) is attached to an ethics committee approved by the French Ministry of Education and Research. No special authorization was required in the frame of the project given the low frequency and volume of blood collections.

### Mosquito populations

Two Culicidae species from Guadeloupe were used for the experiments: *Ae. aegypti* and *Cx. quinquefasciatus*. *Ae. aegypti* individuals were collected in 2018 at larval or pupal instar in artificial breeding-sites located in Lauricisque (16°15′01″N; 61°32′51″W), while *Cx. quinquefasciatus* were collected as eggs rafts in artificial breeding-sites used as ovitraps at the Institute Pasteur of Guadeloupe (16°53′19″N; 61°31′41″W). Both species were collected from 4 to 6 breeding sites and reared under controlled conditions in dechlorinated tap water at 27°C and fed with brewer's yeast capsules since egg hatching. Larvae were split into groups of 150–200 larvae per litre of water. Water and food were renewed every 2–3 days until adult emergence. After emergence, adults were kept in flight rearing cages under controlled conditions (27 ± 1°C; 70% RH; 12:12 h L:D photoperiod) and fed with 10% sucrose solution *ad libitum* until their use in experiments. When necessary, an artificial blood meal using rabbit blood and a hemotek feeding system (Hemotek LTD) was provided to mosquitoes to obtain progeny. The first generation of *Ae. aegypti* mosquitoes (F_1_) was used in the experiments, whereas *Cx. quinquefasciatus* adults issued from eggs collected on the field were directly used (F_0_).

### Viral strains

Three ZIKV strains were used for mosquito oral infections (Table 1). All viral strains were provided as lyophilisates by the Emergence Virus Unit (Marseille) via the initiative “European Virus Archive goes global” (EVAg) (Table 1). Lyophilisates were re-suspended into DMEM medium (Gibco, Fisher scientific, UK) for viral production in Vero cells (ATCC, ref. CCL-81). Viral productions used a multiplicity of infection of 0.1, DMEM medium supplemented with 2% fetal bovine serum (FBS; Gibco, Fisher scientific) and were grown for three days. The viral stocks obtained were kept at −80°C prior to their use in experiments. The viral titer of stocks was estimated by serial 10-fold dilutions on Vero cells and expressed in tissue culture infectious dose 50 (TCID_50_)/ml.

**Table 1.** Characteristics of the virus strains used in this study.

Zika virus strain^a^	Origin	Year of first isolation	Viral stock passage history^b^	% nucleotide relatedness			GenBank Accession number
				Senegal	Martinique	Malaysia	
Senegal	African	1984	P5	–	–	–	KU955592
Martinique	American	2015	P4	88,91	–	–	KU647676
Malaysia	Asian	1966	P5	90,35	95,56	–	KX694533

^a^Supplier strain names: Senegal: Dak84; Martinique: MRS_OPI_Martinique_PaRi_2015; Malaysia: MAS66.

^b^Vero cells (ATCC, Ref.CCL – 81) were used for all the virus passages.

### Mosquito oral infections

Young mated *Ae. aegypti* and *Cx. quinquefasciatus* females (7–10 days old) were respectively starved 24 and 48 h before oral infection. Mono-infected blood meals were prepared with the three viral strains with 1.4 ml of washed rabbit erythrocytes and 700 µl of viral suspension supplemented with the phagostimulant adenosine triphosphate (Sigma-Aldrich, Germany) at a final concentration of 5 mM. The blood meal titer was 10^7^ TCID_50_/ml for each viral strain and was verified from blood aliquots collected before the oral infection and one hour after via TCID_50_ assays. Mosquito feeding was performed with a Hemotek system and was limited to 60 min to avoid any significant decrease of blood meal titer. After the infectious blood meal, the non-engorged females were discarded and the fully engorged were transferred in cardboard containers and maintained with 10% sucrose at 28° ± 1°C for further analysis.

### Infection, dissemination and transmission analysis

For each combination of mosquito population and viral strain, 20–30 females were processed at 7, 14 and 21 days post-infection (dpi). The body of mosquitoes was used to estimate the viral infection, the head was used to assess viral dissemination beyond the mosquito midgut, and the saliva was collected to evaluate the mosquito transmission potential. Saliva collection was conducted as described in Dubrulle and colleagues [23]. Briefly, legs and wings were removed from individual mosquitoes and the proboscis was inserted into a 20-µl tip containing 5 μl of FBS for 30 min. Saliva samples were then expelled into 45 µl of DMEM medium and kept at −80°C before analysis. After salivation, the head was separated from the rest of the mosquito body (abdomen and thorax). Both compartments, bodies and heads, were separately grounded with glass beads in 300 μl of DMEM medium supplemented with 2% FBS and 1X of Antibiotic-Antimycotic (Anti–Anti; Gibco, Fisher scientific). For each combination of mosquito population, viral strain and dpi, the infection rates (IRs), dissemination rates (DRs), dissemination efficiencies (DEs), transmission rates (TRs) and transmission efficiencies (TEs) were calculated. IR refers to the proportion of mosquitoes with infected body among tested ones. DR corresponds to the proportion of mosquitoes with the infected head among those having an infected body. TR represents the proportion of mosquitoes with infectious saliva among mosquitoes with disseminated infection. DE and TE refer to the proportion of mosquitoes with infectious viral particles in the head or in the saliva, respectively, among all tested ones. Saliva titers were also estimated.

### Samples titration

Infectious status of bodies and heads were estimated by a TCID_50_ assay (96-well plates), while saliva infection status and titers were evaluated by a plaque assay (6-well plates). Ten-fold dilutions of samples were conducted in DMEM medium supplemented with 2% FBS, 1X Anti–Anti and inoculated onto Vero cells. Cells were incubated at 37°C, under a 5% CO_2_ concentration for 7 days. For the plaque assays, cells were incubated under an agarose overlay as described in Arias-Goeta and colleagues [24]. Cell monolayers were fixed 7 days after inoculation with a solution of 10% formalin, 0.2% crystal violet and 20% ethanol for 30 min to reveal the cytopathogenic effect.

### Statistical analysis

IR, DR, TR, DE and TE were compared for each combination of mosquito population, viral strain and dpi using Fisher's exact tests. In addition, DE and DR, as for TE and TR, were compared using Fisher's exact tests within each mosquito population, viral lineage and dpi. If multiple Fisher's exact tests were applied to the same data set, then the significance level for each test was adjusted by the sequential Bonferroni method to accommodate the multiple tests. Saliva titers were compared when necessary with non-parametric Kruskal–Wallis test. All statistical tests were conducted using R V. 3.3.2 [25].

## Results

### *Culex quinquefasciatus* mosquitoes from Guadeloupe are not able to experimentally transmit ZIKV

To assess whether *Cx. quinquefasciatus* mosquitoes from Guadeloupe are able to experimentally transmit ZIKV, mosquitoes were orally mono-infected with three ZIKV strains (Senegal, Malaysia and Martinique) at 10^7^ TCID_50_/ml and processed further to determine IR, DR, DE, TR and TE. No infection, nor dissemination nor transmission was detected for any of the ZIKV strains at 7, 14 or 21 dpi (Table 2).

**Table 2.** Infection rates, dissemination rates, and transmission rates of *Culex quinquefasciatus* and *Aedes aegypti* mosquitoes from Guadeloupe at 7, 14 and 21 days post-infection, calculated for three different ZIKV strains. Blood meal titer was 10^7^ TCID_50_/ml for all the experiments. Values in bold correspond to rates significantly different between ZIKV strains, mosquito species and days post-infection (Fisher's exact test, *P* < 0.0001).

Mosquito species	ZIKV strains	Infection rate (%)			Dissemination rate (%)			Transmission rate (%)		
		Day post infection			Day post infection			Day post infection		
		7	14	21	7	14	21	7	14	21
*Culex quinquefasciatus*	Senegal (African)	0	0	0	0	0	0	0	0	0
* *		*(30)*	*(30)*	*(28)*	*(30)*	*(30)*	*(28)*	*(30)*	*(30)*	*(28)*
* *	Malaysia (Asian)	0	0	0	0	0	0	0	0	0
* *		*(13)*	*(25)*	*(23)*	*(13)*	*(25)*	*(23)*	*(13)*	*(25)*	*(23)*
* *	Martinique (American)	0	0	0	0	0	0	0	0	0
* *		*(20)*	*(18)*	*(26)*	*(20)*	*(18)*	*(26)*	*(20)*	*(18)*	*(26)*
*Aedes aegypti*	Senegal (African)	**90**	**92**	**84.6**	**96.3**	**91.3**	**95.5**	42.3	61.9	76.2
* *		*(30)*	*(25)*	*(26)*	*(30)*	*(25)*	*(26)*	*(30)*	*(25)*	*(26)*
* *	Malaysia (Asian)	23.3	23.3	16.7	28.6	71.4	80	0	20	75
* *		*(30)*	*(30)*	*(30)*	*(30)*	*(30)*	*(30)*	*(30)*	*(30)*	*(30)*
* *	Martinique (American)	23.3	36.7	26.7	28.6	54.5	62.5	0	0	80
* *		*(30)*	*(30)*	*(30)*	*(30)*	*(30)*	*(30)*	*(30)*	*(30)*	*(30)*

Note: Numbers in parentheses correspond to the number of analysed mosquitoes.

### Better transmission of ZIKV strain from Senegal by *Ae. aegypti* from Guadeloupe compared to Malaysia and Martinique strains

**Figure 1.**
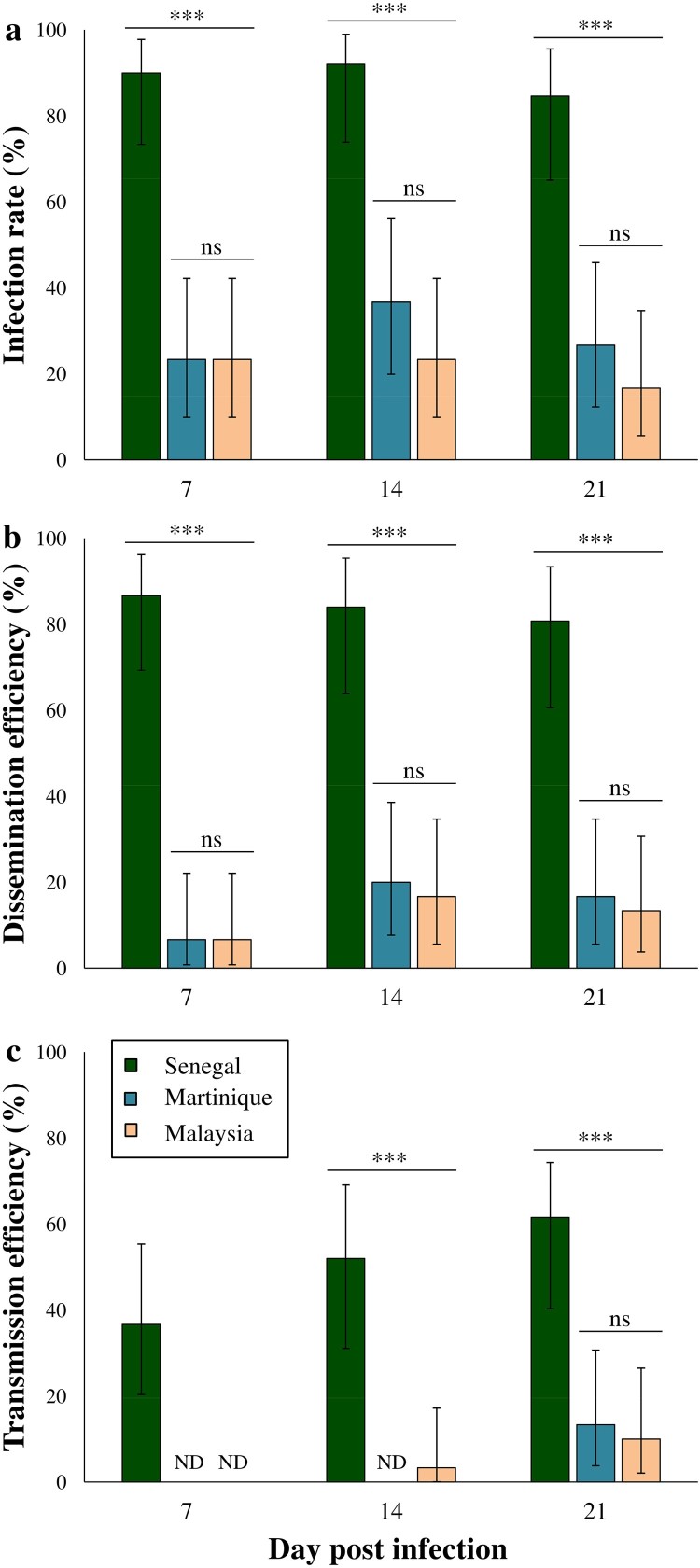
Infection rate (± CI) (a), dissemination efficiency (± CI) (b), and transmission efficiency (± CI) (c) of *Aedes aegypti* from Guadeloupe fed with ZIKV at 7, 14 and 21 days post-infection (dpi). For each modality, a batch of 30 mosquitoes was analysed except for African ZIKV at 14 dpi (*N* = 25) and 21 dpi (*N* = 26). Asterisks indicate significant differences between the estimated parameters according to ZIKV strains and dpi (Fisher's exact test; ***: *P* < 0.0001). ND: Not Detected.

**Figure 2.**
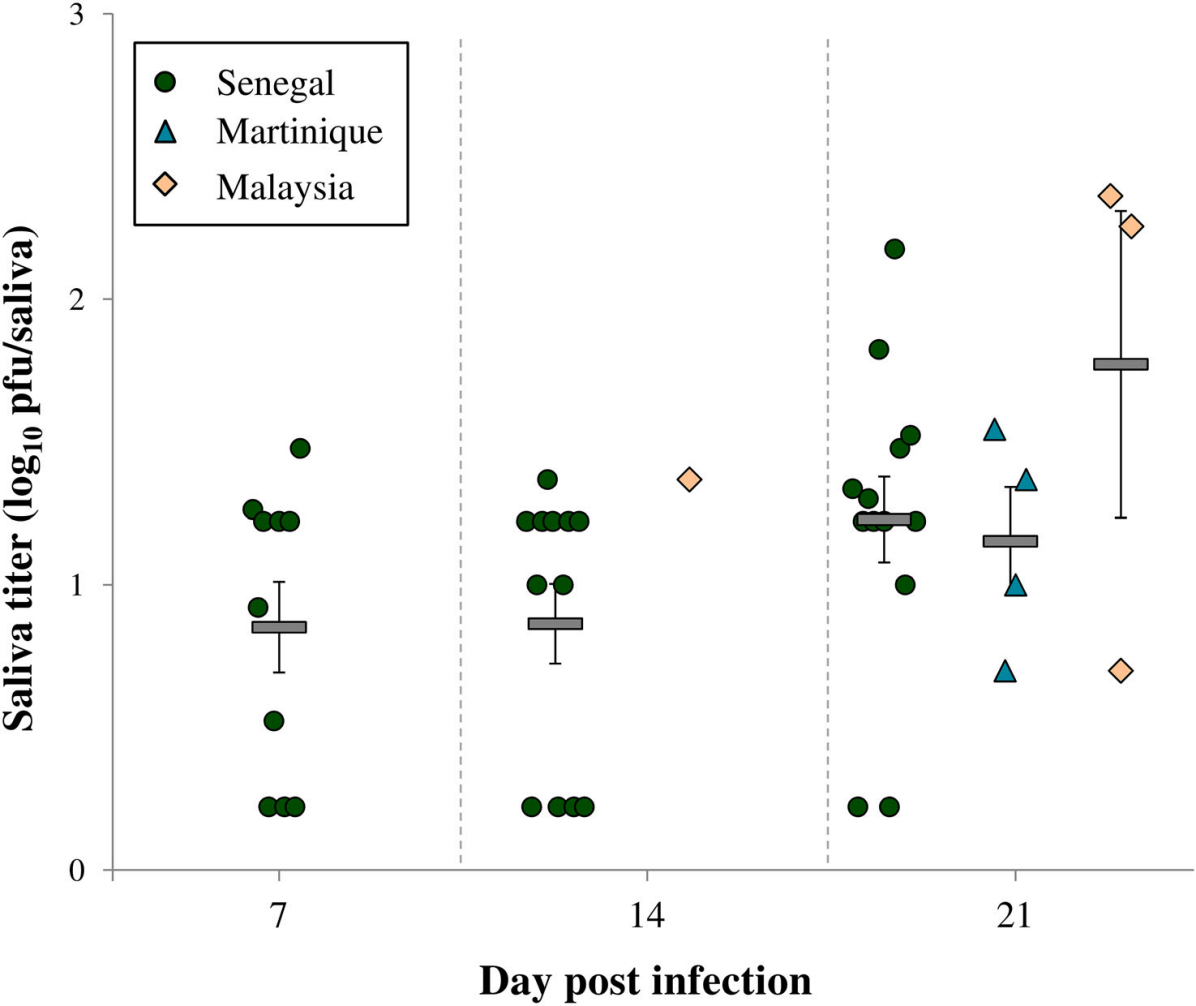
Viral loads in saliva of *Ae. aegypti* infected with ZIKV Senegal, ZIKV Martinique or ZIKV Malaysia at 7, 14 and 21 days post-infection. Dots, triangles and rhombuses, represent the titer of each infected saliva. Horizontal grey bars represent means (± SE).

Vector competence parameters obtained with the viral strains from Senegal, Malaysia and Martinique in *Ae. aegypti* mosquitoes from Guadeloupe were compared at 7, 14 and 21 dpi. The Senegal strain showed significant higher IRs, DRs, DEs and TEs when compared to strains from Martinique and Malaysia whatever the day post-infection (Fisher's exact tests; *P* < 0.0001 for all the parameters). Indeed, the mean IRs, DRs, DEs and TEs recorded for Senegal strain were respectively 88%, 94%, 83% and 50%, while the averages for these parameters in Malaysia and Martinique strains were 25%, 54%, 13% and 12% (Figure 1; Table 2). The EIP also differed between viral strains, with ZIKV detected in mosquito saliva since 7 dpi for Senegal strain, 14 dpi for Malaysia strain and 21 dpi for Martinique strain (Figure 1). The overall TRs ranged between 20% and 80% but no significant differences were observed for the three viral strains at 21 dpi (Fisher's exact test; *P* = 0.206) (Table 2). Regarding the Asian/American strains, ZIKV from Martinique exhibited slightly superior IRs, DEs and TEs when compared to Malaysia strain, but these differences were not statistically significant whatever the day post-infection (Fisher's exact tests; *P* > 0.05). The mean ZIKV viral loads detected in *Ae. aegypti* saliva ranged between 0.85 ± 0.16–1.77 ± 0.54 log_10_ pfu/saliva and were similar for all viral strains (Kruskal–Wallis test; *P* > 0.05) (Figure 2). At 21 dpi, the mean saliva titer was higher with ZIKV Malaysia when compared to Senegal and Martinique strains, but this difference was not statistically significant (Kruskal–Wallis test; *P* = 0.417).

## Discussion

To date, vector control is the best prospects for controlling ZIKV. Thus, it is important to know the mosquito species that are implicated in ZIKV transmission in Guadeloupe to properly apprehend the epidemiological risk on the island and adapt vector control strategies. The current vector control methods, only focusing on *Ae. aegypti*, may be not suitable to control ZIKV if another local species with different biological characteristics, ecology and host-related behaviours is involved in the transmission of the virus. In this study, we focused on the mosquito species *Cx. quinquefasciatus* for several reasons: (i) they are anthropophilic [26] and ubiquitous in urban areas of Guadeloupe [16], (ii) they are vectors of flaviviruses such as West Nile virus and St-Louis encephalitis virus [17] and (iii) recent scientific literature recognizes *Cx. quinquefasciatus* as a potential ZIKV vector, based either in vector competence experimental assays and field investigations [5,13,21,22,27]. Three of these studies have even found infectious ZIKV particles in saliva or salivary glands of this mosquito species after artificial infections.

In our study, the results obtained from the 213 tested mosquitoes show a complete refractoriness of *Cx. quinquefasciatus* from Guadeloupe to ZIKV infection and subsequently, neither dissemination nor transmission was detectable whatever the strain tested. Under the experimental conditions used for this vector competence assay, ZIKV was not able to infect the mosquito body. These findings are in agreement with the majority of published vector competence studies, where *Cx. quinquefasciatus* was also found to be refractory to the infection whatever the ZIKV lineage used [20,28–32]. In these previously-cited studies, the refractoriness to ZIKV infection has been generally evaluated until 14 days after blood meal ingestion. Our research presented herein showed that even after 21 dpi, the mosquitoes are still resistant to ZIKV infection. The refractoriness of *Cx. quinquefasciatus* midgut epithelial cells to ZIKV infection may be explained by a protein incompatibility between the virus and the midgut cell receptors [33]. However, refractoriness to ZIKV transmission has also been detected in *Cx. quinquefasciatus* mosquitoes infected intrathoracically [34], which suggests that mosquito immunity and/or the salivary gland barrier can also prevent ZIKV transmission in this mosquito species. The high *Wolbachia* infection in *Cx. quinquefasiatus* from Guadeloupe [35] has been also suspected to confer refractoriness to ZIKV, but experimental studies showed that even when *Wolbachia* is removed from this mosquito species, ZIKV fails to infect the midgut epithelial cells [20]. Taken together, these results show that *Cx. quinquefasciatus* from Guadeloupe is not involved in ZIKV transmission and therefore, the vector control strategies implemented locally for this virus should not focus on this mosquito species. As the genotypes of arboviruses and their vectors, as well as their interaction are primordial to assess the vector competence for a given pathogen [4,36,37], we used in this study three ZIKV strains (American, Asian and African) to comprehensively estimate the different levels of vector competence that can be displayed by *Ae. aegypti* from Guadeloupe. Our results showed that this local population is able to transmit ZIKV, with a vector competence that varies considerably depending on the ZIKV strain considered. *Ae. aegypti* was the major ZIKV vector during the outbreaks that occurred in 2016 in the Americas and the West Indies, including Guadeloupe. The vector competence of *Ae. aegypti* from Guadeloupe was already assessed once using a ZIKV strain from Asian lineage isolated in New Caledonia, but no transmission was confirmed neither at 4 nor at 7 dpi [3]. Here, we used another Asian strain isolated in Malaysia and we assessed the vector competence at later days post-infection (7, 14 and 21 dpi), which allowed us to confirm the competence of these mosquitoes regarding Asian ZIKV strains despite the low IRs and DRs obtained with respect to Chouin-Carneiro and colleagues (2016) [3] (Table 2; Figure 1). No transmission was detected at 7 dpi for the Asian strain from Malaysia as previously found with the New Caledonia strain [3]. However, transmission was detected after a greater EIP, at 14 dpi and increased by 21 dpi.

The American strain used circulated in Martinique during the explosive West Indies outbreaks and has 99.56% nucleotide sequence identity with the strains that circulated in Guadeloupe (Table 1). Surprisingly, low IRs, DRs, DEs, TRs, TEs were recorded with this virus and the EIP was the longest of all the tested strains with transmission detected only at 21 dpi. This low vector competence for the Martinique strain in *Ae. aegypti* from Guadeloupe was unexpected due to the rapid spread of the disease through the island in the absence of another vector. However, it is known that artificial blood meals may reduce the infection rates when compared to a mammal model [38,39], meaning that the estimated rates obtained with our experimental approach can be underestimated for the species. Regarding EIP, more than 14 days are needed for the ingested virus to reach the mosquito saliva, which implies that mosquito should survive more than two weeks before they can propagate the infection into a new host. Optimal environmental circumstances [40] and high mosquito densities may have counteracted the low vector competence of local populations and favoured their vectorial capacity during the critical phase of the outbreak. However, entomological investigations in the field and exhaustive analysis of climatic data are still lacking to confirm this hypothesis.

The feeding habits of *Ae. aegypti* could also have increased its vectorial capacity, as these mosquitoes often feed on humans with multiple bites in a single gonotrophic cycle [41]. A single infected mosquito has thus the potential to infect rapidly several humans during a short period of time [42].

The highest vector competence and the shortest EIP were obtained in *Ae. aegypti* mosquitoes infected with the African ZIKV strain when compared to the Asian and American ones. Indeed, we demonstrated that *Ae. aegypti* from Guadeloupe were able to transmit the Senegal strain since 7 dpi with a TE of 36% and by 21 dpi, 61% of mosquitoes had ZIKV in their saliva (Figure 1). The high IRs, DRs and DEs recorded with the Senegal strain suggest the great capability for this virus to bypass both the intestinal and salivary gland barriers in this mosquito, while for Asian and American strains the midgut barrier and mosquito immunity may have importantly decreased dissemination success as witnessed by the low IRs and Des [43] (Figure 1). However, once the virus passed the midgut barrier, the probability to reach the saliva was high for the three ZIKV strains and the viral loads reached were similar (Table 2; Figure 2), hence showing the permissiveness of the salivary gland barrier.

The results presented above highlight differences in *Ae. aegypti* vector competence according to the viral strain used and are in agreement with previous studies. Indeed, even if Pacific, American and Caribbean ZIKV outbreaks involved strains related to the Asian lineage, the infection levels and vector competence displayed by *Ae. aegypti* from these epidemic regions are higher for African ZIKV strains [12,38,44,45]. Our results reinforce the hypothesis that African ZIKV lineage is more infectious for *Ae. aegypti* populations from epidemic regions outside Africa than for African *Ae. aegypti* populations [12,46]. Consequently, the evolutionary hypothesis proposing that the recent emergences of ZIKV Asian strains were favoured by a ZIKV adaptation for higher vector competence and enhanced horizontal transmission of the virus by epidemic vectors, as it has been the case for chikungunya virus and *Ae. albopictus* mosquitoes [47], would not be plausible.

In conclusion, this vector competence study confirmed that *Ae. aegypti* from Guadeloupe can transmit ZIKV strains belonging to Asian, African and American lineages. The African ZIKV strain ensured the highest vector competence levels and the shortest EIP when compared to the Asian and the American strains which showed unexpected low vector competence in local *Ae.aegypti*. *Cx. quinquefasciatus* from Guadeloupe are refractory to experimental ZIKV infection and are likely not able to ensure an autochthonous transmission of the virus in the field. Therefore, vector control efforts must continue to focus on reducing the urban populations of *Ae. aegypti* in Guadeloupe.
